# 706 Epidemiology, Therapeutic Complexity and Prognostic Challenges of Burns to the Perineum, Buttocks and Genitals

**DOI:** 10.1093/jbcr/irad045.182

**Published:** 2023-05-15

**Authors:** Gabriel Hundeshagen, Bastian Bonaventura, Alexander Runkel, Christian Tapking, Valentin Haug, Kamal Hummedah, Ulrich Kneser

**Affiliations:** BG Unfallklinik Ludwigshafen, University of Heidelberg, Ludwigshafen, Rheinland-Pfalz; BG Klinik Ludwigshafen, Ludwigshafen, Rheinland-Pfalz; Klinik für Plastische und Handchirurgie,Universitätsklinikum Freiburg, Freiburg, Baden-Wurttemberg; BG Unfallklinik Ludwigshafen, University of Heidelberg, Ludwigshafen, Rheinland-Pfalz; BG Unfallklinik Ludwigshafen, University of Heidelberg, Ludwigshafen, Rheinland-Pfalz; BG Unfallklinik Ludwigshafen, University of Heidelberg, Ludwigshafen, Rheinland-Pfalz; BG Unfallklinik Ludwigshafen, University of Heidelberg, Ludwigshafen, Rheinland-Pfalz; BG Unfallklinik Ludwigshafen, University of Heidelberg, Ludwigshafen, Rheinland-Pfalz; BG Klinik Ludwigshafen, Ludwigshafen, Rheinland-Pfalz; Klinik für Plastische und Handchirurgie,Universitätsklinikum Freiburg, Freiburg, Baden-Wurttemberg; BG Unfallklinik Ludwigshafen, University of Heidelberg, Ludwigshafen, Rheinland-Pfalz; BG Unfallklinik Ludwigshafen, University of Heidelberg, Ludwigshafen, Rheinland-Pfalz; BG Unfallklinik Ludwigshafen, University of Heidelberg, Ludwigshafen, Rheinland-Pfalz; BG Unfallklinik Ludwigshafen, University of Heidelberg, Ludwigshafen, Rheinland-Pfalz; BG Unfallklinik Ludwigshafen, University of Heidelberg, Ludwigshafen, Rheinland-Pfalz; BG Klinik Ludwigshafen, Ludwigshafen, Rheinland-Pfalz; Klinik für Plastische und Handchirurgie,Universitätsklinikum Freiburg, Freiburg, Baden-Wurttemberg; BG Unfallklinik Ludwigshafen, University of Heidelberg, Ludwigshafen, Rheinland-Pfalz; BG Unfallklinik Ludwigshafen, University of Heidelberg, Ludwigshafen, Rheinland-Pfalz; BG Unfallklinik Ludwigshafen, University of Heidelberg, Ludwigshafen, Rheinland-Pfalz; BG Unfallklinik Ludwigshafen, University of Heidelberg, Ludwigshafen, Rheinland-Pfalz; BG Unfallklinik Ludwigshafen, University of Heidelberg, Ludwigshafen, Rheinland-Pfalz; BG Klinik Ludwigshafen, Ludwigshafen, Rheinland-Pfalz; Klinik für Plastische und Handchirurgie,Universitätsklinikum Freiburg, Freiburg, Baden-Wurttemberg; BG Unfallklinik Ludwigshafen, University of Heidelberg, Ludwigshafen, Rheinland-Pfalz; BG Unfallklinik Ludwigshafen, University of Heidelberg, Ludwigshafen, Rheinland-Pfalz; BG Unfallklinik Ludwigshafen, University of Heidelberg, Ludwigshafen, Rheinland-Pfalz; BG Unfallklinik Ludwigshafen, University of Heidelberg, Ludwigshafen, Rheinland-Pfalz; BG Unfallklinik Ludwigshafen, University of Heidelberg, Ludwigshafen, Rheinland-Pfalz; BG Klinik Ludwigshafen, Ludwigshafen, Rheinland-Pfalz; Klinik für Plastische und Handchirurgie,Universitätsklinikum Freiburg, Freiburg, Baden-Wurttemberg; BG Unfallklinik Ludwigshafen, University of Heidelberg, Ludwigshafen, Rheinland-Pfalz; BG Unfallklinik Ludwigshafen, University of Heidelberg, Ludwigshafen, Rheinland-Pfalz; BG Unfallklinik Ludwigshafen, University of Heidelberg, Ludwigshafen, Rheinland-Pfalz; BG Unfallklinik Ludwigshafen, University of Heidelberg, Ludwigshafen, Rheinland-Pfalz; BG Unfallklinik Ludwigshafen, University of Heidelberg, Ludwigshafen, Rheinland-Pfalz; BG Klinik Ludwigshafen, Ludwigshafen, Rheinland-Pfalz; Klinik für Plastische und Handchirurgie,Universitätsklinikum Freiburg, Freiburg, Baden-Wurttemberg; BG Unfallklinik Ludwigshafen, University of Heidelberg, Ludwigshafen, Rheinland-Pfalz; BG Unfallklinik Ludwigshafen, University of Heidelberg, Ludwigshafen, Rheinland-Pfalz; BG Unfallklinik Ludwigshafen, University of Heidelberg, Ludwigshafen, Rheinland-Pfalz; BG Unfallklinik Ludwigshafen, University of Heidelberg, Ludwigshafen, Rheinland-Pfalz

## Abstract

**Introduction:**

Burns that involve the perineum, buttocks and genitals (PBG) have been associated independently with more challenging therapeutic needs and worse clinical outcomes in case series and smaller patient collectives, while large scale epidemiology of this injury is lacking. We studied a cohort of 1024 patients admitted to our burn center to assess clinical outcomes based on the presence of PBG burns.

**Methods:**

Medical records were screened for patients who did or did not sustain PBG burns and patients stratified accordingly (PBG vs. control (CTR)). Demographic baseline data, burn etiology, presence of inhalation injury (IHT), presence of full thickness burns (FT), number of operations (NOR), mortality, length of ICU treatment (LOS ICU) and hospitalization (LOS) were assessed to compare clinical characteristics and outcomes between the groups. Multivariate regression analyses adjusting for TBSA, sex and age were conducted for key clinical outcomes.

**Results:**

Between 2014 and 2022, 1024 patients were included in the analysis (PBG: n=227; CTR: n=797). PBG burns were older (median (IQR) 54 (38) vs. 44, (31) years, p< 0.0001), more frequently female (35% vs. 23%, p=0.002) presented with larger TBSA burns overall (27 (37) vs. 10 (13) %, p< 0.0001) and sustained FT burns more frequently (69% vs. 26% p< 0.0001). Scald burns were more frequently the cause of PBG burns (45% vs. 15%, p< 0.0001), PBG patients needed twice as many surgical procedures (2 (4) vs. 1 (1), p< 0.0001) as CTR. PBG patients remained longer in the ICU per per cent TBSA (0.9 (1.1) vs. 0.5 (0.8) days/%TBSA, p< 0.001). Mortality was higher in patients with PBG burns (33.1% vs. 7.8%, p< 0.0001). Multivariate logistic regression yielded significant effects of PBG burns on LOS ICU, LOS and NOR.

**Conclusions:**

Burns to the perineum, buttocks and genitals are associated with more challenging baseline characteristics upon admission and substantially worse clinical outcomes than burns not involving PBG.

**Applicability of Research to Practice:**

Expectable therapeutic challenges regarding prolonged intensive care, more frequent surgeries and increased risk for mortality should be taken into account and planned for when admitting patients with PBG burns.

